# Chromosomal 1p Duplication in a Pediatric Patient: A Case Report

**DOI:** 10.7759/cureus.64911

**Published:** 2024-07-19

**Authors:** Arthur Pavlovsky, Camryn R Marshall, Savannah Braud, Everett J Kim, Mario Jacomino

**Affiliations:** 1 Medicine, Florida Atlantic University Charles E. Schmidt College of Medicine, Boca Raton, USA; 2 Women's and Children's Health, Florida Atlantic University Charles E. Schmidt College of Medicine, Boca Raton, USA

**Keywords:** delayed development, 1p31.3, delayed developmental milestones, intellectual disability, feeding difficulty, failure to thrive, chromosome 1p duplication

## Abstract

Chromosomal 1p duplications are a rarity, with minimal literature on the topic. As a result, it is useful to document patient presentations with this defect to help guide the management and treatment of future patients with this genetic abnormality. We present a successful case report of a patient with a chromosome 1p31.3p31.1 duplication, including her initial presentation, the path to genetic testing, and patient outcome. Chromosomal duplication was found on genetic testing performed for failure to thrive and inability to meet her developmental milestones. The patient was significantly undernourished due to her feeding difficulties, leading to her presentation of altered mental status, growth arrest, dehydration, and hypoglycemia. Intervention in the form of a gastrostomy tube and fundoplication led to a significant improvement in the stability seen in the patient at the time of discharge. Long-term cognitive-linguistic treatment is required for continued neurological development. Only 11 publications currently exist regarding chromosome 1p duplication. However, none are specific to the 1p31.3p31.1 duplication, making this case report the first of its kind. Overlapping chromosomal 1p duplications have been described in patients with low birth weight and growth delays, palate abnormalities, intellectual disability, microcephaly, heart defects, and ambiguous genitalia. Despite the rarity of this duplication, it is essential to document these cases because if some of these genetic abnormalities are identified in more significant numbers, they can be conclusively linked to the patient’s phenotype. In addition, the treatment plan played an instrumental role in stabilizing our patient's condition. It is also helpful to report the treatment plans so future clinicians who encounter this situation can utilize the successful treatment plans that most align with their patient’s clinical presentation.

## Introduction

Chromosome 1 is the largest of the human chromosomes. Several diseases are associated with disruptions in the sequence of this chromosome, including cancers, neurological and developmental disorders, and Mendelian conditions [1]. The availability of high-resolution chromosomal microarrays and whole-exome sequencing makes it easier to conduct genetic analysis to detect microscopic deletions or duplications that could be associated with neurogenetic syndromes. Several susceptibility regions of chromosome 1 have been identified with known genetic syndromes, such as the 1p36 region [1]. The subchromosomal region 1q21.1 is also a hotspot for deletions and duplications, which manifest phenotypic abnormalities that have been previously identified in the literature, including 1q21.1 deletion and duplication syndromes or thrombocytopenia-absent radius syndrome [2,3]. A recent study documented that multiple rare variants are also present in other susceptible regions of chromosome 1 [4]. So, reporting these variants is essential to update databases and literature, allowing for better diagnosis and genetic counseling [4]. Because the presence of these variants in the patient’s genetic composition may show variability in their phenotype, reporting will also help establish phenotype-genotype correlations to identify specific genetic syndromes.

Genetic analyses are a valuable reference for guiding the assessment and treatment of patients with dysmorphic features, abnormal physical exam findings, or developmental delays. In cases of chromosomal 1p duplication, however, research is limited due to the scarcity of this defect. After a thorough literature review, only 11 other publications exist regarding chromosomal 1p duplications [5]. The varying cases reported highlight the diverse nature of the phenotypical presentation that this duplication provides. Here, we present a case study of a two-year-old female with a 1p31.3p31.1 duplication to help guide future treatment of individuals with this defect.

## Case presentation

A two-year-old female presented to a local hospital with behavior altered from her baseline, according to her mother. She was sleepy but responsive to pain stimuli. She was an only child, born at 3.18 kg via C-section due to a previous maternal C-section after an uncomplicated pregnancy and uneventful labor and delivery. She did not require any resuscitation measures at birth. The mother could not confirm a family history of developmental delay. The child had significant developmental delays, including delayed walking and talking and a lack of weight gain for one year. Her physical exam revealed dehydration, rhinorrhea, dry and cracked lips, and pale mucous membranes. Her neurologic exam showed considerable weakness in all extremities and an inability to lift her head or sit on her own. According to her mother, the patient was delayed in language development and had not spoken her first words. She also exhibited significant joint laxity during the passive range of motion. Her admission weight was 9.9 kg (third percentile for age), her length was 79 cm (third percentile for age), and her head circumference was 48.5 cm (90th percentile for age). A complete bone survey suggested growth arrest, and a head CT without contrast was initially interpreted as a subarachnoid hemorrhage of unclear etiology. She was transferred to another hospital’s pediatric trauma unit for further management.

**Figure 1:**
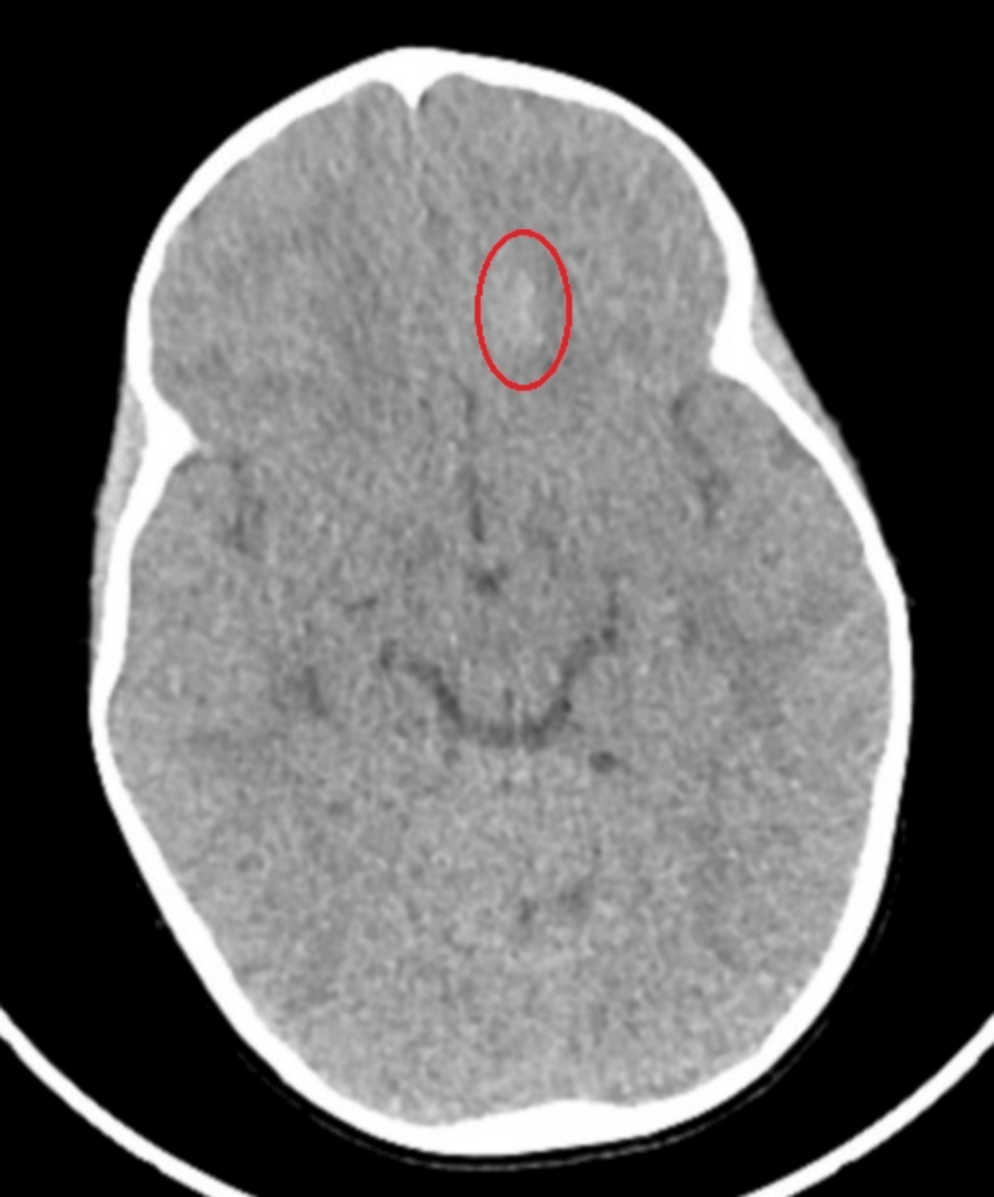
Non-contrast CT scan of the head: axial slice at the level of the third ventricle The left frontal lobe shows an ill-defined ovoid focus of subtle hyperdensity (red circle) without mass effect or surrounding edema. This finding is asymmetric compared to the right lobe, and no other abnormalities are detected in the rest of the brain parenchyma. This is a nonspecific finding. Differential considerations include artifacts, calcification from a remote insult or underlying mass, or, less likely, age-indeterminate blood products. An MRI would be warranted if there is a concern about bleeding.

At the pediatric trauma hospital, a thorough review of the CT scan revealed nonspecific cerebral calcification rather than intracranial hemorrhage (Figure 1). The patient was cleared for discharge from a pediatric neurosurgery perspective and transferred to the pediatric intensive care unit (PICU) at a third hospital due to her altered mental status. An MRI of the brain was not performed as it was not expected to alter the course of management.

Compensated metabolic acidosis was found on initial examination at the PICU of the third hospital and was corrected with IV sodium bicarbonate. The patient was lethargic with a severe aversion to feeding and food in her mouth. She appeared unable to swallow, with noticeable saliva retention. Total parenteral nutrition was administered via a central line through the right internal jugular vein, later switched to PediaSure® via a nasoduodenal tube. She was also found to have hypertrophic tonsils and adenoids, along with laryngomalacia, causing intermittent, brief inspiratory stridor, which resolved without treatment.

At the time of admission to the third hospital, the patient’s growth delay suggested a metabolic or genetic disorder, leading to the performance of a whole genome single nucleotide polymorphism microarray from a blood specimen. This study revealed an interstitial duplication of chromosome 1p, specifically segment 1p31.3p31.1. (1 = chromosome, p = short segment of chromosome 1, p31.3p31.1 = genomic interval on chromosome 1p from sub-band 31.3 to sub-band 31.1). Parental bacterial artificial chromosome fluorescence in situ hybridization (BAC-FISH) analysis was recommended to confirm whether the alteration was a de novo change and to rule out a balanced rearrangement with a high reproductive risk. However, this analysis has yet to be performed. Initial arterial blood gases revealed metabolic acidosis, which was corrected with IV sodium bicarbonate. A chest X-ray was unremarkable. The patient underwent Holter monitoring for abnormal cardiac rhythm, which only detected premature atrial contractions. Cardiac echocardiography was unremarkable, showing no evidence of heart disease. An electroencephalogram was also performed and was read as normal.

Several swallowing studies were unsuccessful due to the patient’s inability and refusal to swallow. After one week of tube feedings, a swallow study was finally performed and read as negative for aspiration or abnormalities of the upper esophagus or achalasia. Throughout her hospitalization, the patient continued to refuse oral feedings. On several occasions, the patient’s mother declined both gastric (G) and gastrojejunostomy tube placement. Further discussion with the patient’s mother regarding the patient’s outcomes led to the decision to place a G-tube with Nissen fundoplication due to concerns about gastroesophageal reflux and the increased risk of aspiration. Before the placement of the G-tube and fundoplication, the patient was found to have severe iron deficiency anemia, requiring packed red blood cell transfusion due to a hemoglobin level of 7 g/dl and a hematocrit of 22%. The patient was placed on oral supplemental iron. Gastroenterology was consulted to manage the patient’s G-tube.

The patient received occupational, physical, and speech therapy, which was initiated two weeks into her hospitalization. Her neurological status improved rapidly, and at the time of discharge, she was awake, alert, and interacting cheerfully with her environment. She had a well-healed gastrotomy scar and severe laxity of joints in all extremities. Her laboratory test results, including a comprehensive metabolic panel, serum amino acids, carnitine, free fatty acids, lactate, pyruvate, thyroid function, hepatic panel, urine organic acids, urinalysis, urine culture, and blood culture, were within normal limits. Although she had not yet developed speech, she held a children’s book while smiling and cooing. After one month of inpatient care, the patient was discharged with a weight of 11.2 kg (25th percentile for age) and a head circumference of 49.5 cm (95th percentile for age). She was advised to follow up with both surgery and gastroenterology, and she was provided guidance on G-tube feeds and iron supplementation.

## Discussion

Our case report presents our observations of a two-year-old patient with diverse and complex symptoms that may be linked to a chromosomal abnormality. Presenting symptoms revealed language delay, failure to thrive due to feeding difficulties, dehydration, metabolic acidosis, laryngomalacia, and severe laxity of joints. The patient’s inability to thrive and severe growth delay suggested a metabolic or genetic disorder. The patient’s whole genome microarray revealed an interstitial duplication of chromosome 1p, specifically segment 1p31.3p31.1, the short arm of chromosome 1.

Duplications in specific segments on chromosome 1, specifically in the sub-sections 31.3 to 31.1, are rare. In a thorough literature search, only 11 publications studied chromosome 1p duplications [5]. Across studies, common presentations seen in patients with duplication regions of chromosome 1p included growth retardation, congenital heart defects, metopic synostosis, developmental delay, ambiguous genitalia, and early mortality [6]. The diverse nature of these presentations suggests further study of the complexity and differences in our patient’s symptoms. Interstitial duplications involving the short arm of chromosome 1, however, do commonly present with various phenotypes, as one possibility is the difference in afflicted genes.

One significant commonality between studies shows delayed milestones during infancy among patients with a chromosome 1p duplication. Lennon et al. presented a case of a five-year-old Caucasian female patient diagnosed with a duplication in segment 1p34.134.3 who presented with congenital heart defects and dysmorphic features [5]. The patient, however, presented a similar history of delays in speech and language development at two years of age, which matches the age of our patient. Wieacker et al. presented a patient with a duplication of 1p22.3p32.3 that had growth retardation and developmental delay as our patient had, although those were the only similarities found [7]. Lo et al. shared a similarity with our patient, as theirs had joint laxity [8].

Across studies on chromosomal 1p duplication, there is variability in the demographics of patient age, genetic and family history, presenting symptoms, and the sites of interstitial duplication on chromosome 1. Due to the limitations of obtaining further information on our patient’s family and social history, we cannot assess whether the presenting symptoms may be linked to a family history of poor feeding as an infant or conditions related to the additional presenting symptoms. In Lennon et al.’s study, the five-year-old patient’s chromosomal 1p duplication was the first to be characterized by breakpoint analysis using FISH [5]. Findings showed the patient’s duplication of 1p34.1p34.3 spanned 8.5 MB, marking it as the smallest to contain 1p34 in patients with congenital heart defects [5].

The affected gene interval in our patient is of considerable size, with several genes found on 1p31.3p31.1 that may have contributed to some of our patient’s clinical manifestations. The NEGR1 gene, specifically on 1p31, has been linked to intellectual disability and learning difficulties as it affects neuronal growth, proliferation, and differentiation [9]. The NFIA gene, also on the same 1p31 loci, has been associated with similar outcomes when disrupted [10]. Thakur et al. proposed that several genes, such as LEPR and JAK1, involving 1p31.1-1p31.3, may be relevant in pituitary and overall brain development [11]. Furthermore, most cases evaluated by Thakur et al. had signs of developmental delay [11]. Although these genes have been documented in cases of genetic microdeletion rather than interstitial duplication, we theorize that the possibility of similar outcomes with a duplication could also happen.

This case report is the first to discuss a patient with this permutation of a genetic defect in the 31st genetic interval of the short arm of chromosome 1 and its presenting clinical symptoms. The unique clinical presentation of our patient, compared to the limited cases in the existing literature on 1p duplications, highlights the importance of ongoing surveillance. It is helpful to continue documenting these cases because if some of these abnormalities are identified in a sufficiently large number of patients, they can be conclusively linked to the phenotype, which could be delineated by comparing clinical features among affected individuals [12].

Supportive interventions of nutritional support and speech therapy were instrumental in stabilizing and improving our patient’s condition. This treatment information is also helpful to report so future clinicians who encounter it can utilize the successful treatment plans that most align with their patient’s clinical presentation. As we were unable to obtain a BAC-FISH from the patient and the mother, this limitation further suggests the importance of obtaining information on the patient’s care before the visit, including pre-, peri-, and post-natal care, as well as detailed family and genetic history, in evaluating additional causes or factors. The other important step is long-term follow-up of this patient after discharge, as the mother will need further support and guidance related to the care of the G tube and advancing feedings for the patient as she ages.

## Conclusions

Literature on chromosome 1p duplication is limited. However, a few cases have been described in patients with low birth weight and growth delays, palate abnormalities, intellectual disability, microcephaly, heart defects, and ambiguous genitalia. Despite the rarity of this duplication, it is helpful to document additional case reports like ours so future clinicians who encounter new cases can utilize the successful treatment plans we observed to align with their patient’s clinical presentation. Performing BAC-FISH studies on patients and parents is suggested to further current knowledge on chromosome 1p duplications and identify regions associated with typical symptoms. Continued reporting of cases with chromosome abnormalities will augment our understanding of chromosome 1p duplications and establish phenotype-genotype correlations. The use of new technologies regarding genetic analysis will continue to allow for the routine detection of deletions and duplications of genes. The classification of these deletions and duplications may change over time since genomic information is frequently updated. Future research is warranted as more cases are documented.
